# Human Motion Recognition by Textile Sensors Based on Machine Learning Algorithms

**DOI:** 10.3390/s18093109

**Published:** 2018-09-14

**Authors:** Chi Cuong Vu, Jooyong Kim

**Affiliations:** Department of Organic Materials and Fiber Engineering, Soongsil University, Seoul 156-743, Korea; cuongvc287@gmail.com

**Keywords:** wearables, human motion monitoring, SWCNT, textiles, machine learning algorithm

## Abstract

Wearable sensors for human physiological monitoring have attracted tremendous interest from researchers in recent years. However, most of the research involved simple trials without any significant analytical algorithms. This study provides a way of recognizing human motion by combining textile stretch sensors based on single-walled carbon nanotubes (SWCNTs) and spandex fabric (PET/SP) and machine learning algorithms in a realistic application. In the study, the performance of the system will be evaluated by identification rate and accuracy of the motion standardized. This research aims to provide a realistic motion sensing wearable product without unnecessary heavy and uncomfortable electronic devices.

## 1. Introduction

Wearable technology, especially wearable sensors, has become mainstream these days, and attracted great interest from researchers. By focusing on revealing the multi-dimensional aspects of human life, the wearable tech can be widely applied in medical, healthcare, power sources, flexible electronic components, etc. In the healthcare field, patients can be quickly diagnosed and treated for a variety of diseases with the help of the devices [1]. In sport, athletes’ performance is monitored in order to detect abnormalities, prepare training and tactics plans, or protect them from injuries [2]. Through special structures, wearable electronics can be applied in flexible batteries [3,4], capacitive energy storage [5], data storage [1,2,3,4,5,6], or fashion [7].

Most of the operating mechanisms of sensors are based on a relationship between some physical or chemical quantity such as temperature, pressure, stretch, light, sound, vibration, distance, humidity, pH, and electrical properties such as resistance, electromagnetism, or the capacitance of constituent conductive materials. According to this principle, a popular design approach for wearable sensors is to integrate electronic devices including temperature guage, stretch, proximity, accelerometry, and pulse-oximeter sensors into a small hard packet added on clothes, jewelry [2,8,9,10,11] or directly on the skin [12,13,14]. For example, Son et al. [1] developed bio-integrated systems for diagnosis and therapy of movement disorders. Someya et al. [14] discussed the latest progress in the use of soft electronic materials and their related devices in biological interfaces. Lee et al. [15] studied the development of skin-mounted graphene-hybrid (GP-hybrid) device arrays capable of sweat-based glucose and pH monitoring in conjunction with a sweat-control layer. Gao et al. [16] presented a mechanically flexible and fully integrated sensor array for multiplexed in-situ perspiration analysis, which simultaneously and selectively measures sweat metabolites and electrolytes. Dobkin et al. [17] used gyroscopes, accelerometers, and other physiologic sensors to monitor distance, gait asymmetry, and smoothness of human movements. Wang et al. [18] developed flexible pressure sensors based on polydimethylsiloxane (PDMS) films for monitoring physiological signals. Many other studies on stretch sensors [19,20,21], e-skins [12,13], temperature sensors [21,22], pressure sensors [2,13,23] used graphene [23,24], or carbon nanotubes (CNTs) [11,12,25] as sensing materials. The resulting sensors were effectively proven to be highly sensitive materials good for wearable devices. On the other hand, nanowires (NWs), such as gold NWs [1], Ge/Si-ZnO NWs [18], silver NWs [26,27] or compound mixtures [28] also showed positive results. However, all the above studies have at least one of the following disadvantages: hard electronic components which are inconvenient when feeling or moving, complex fabrication methods or high-cost, or a lack of suitable signal processing algorithms to apply in an actual product.

This research developed a complete combination of the wearable sensor fabrication based on single-walled carbon nanotubes (SWCNT) [11,18,28,29], spandex fabric (PET/SP), and using machine learning algorithms [30] for the analysis of sensing signals in order to apply to the real products in human motion monitoring applications [31,32,33,34,35]. The conductive polyethylene terephthalate (PET/Spandex) fabrics were prepared by padding conductive ink (SWCNT) in order to construct textile fabric sensors. The performance of the fabricated textile sensors has been characterized in terms of their mechanical and electrical performance along with stretch ratio or stretch percentage. Human motion data signals obtained through the e-textile stretch sensor are processed by a specially designed circuit, which digitizes and arranges signals into a custom format to be analyzed further. Then, the data would be transmitted via Bluetooth to the mobile phone [36,37,38,39], tablet or desktop computer in real time for display or analysis based on machine learning algorithms in order to get the best classification of four predefined standardized human motions such as walking, running, sprinting, and jumping.

Machine learning (ML) algorithms have been applied frequently to a variety of fields in medical diagnosis, natural language processing, online searches, smart cars, marketing personalization, etc. In particular, within the field of data analytics, machine learning algorithms are one of the promising methods used to devise complex models that lend themselves to high accuracy prediction and classification tasks. Some useful ML algorithms for classification have been proposed such as random forest (RD) [40], support vector machine (SVM) [41], neural network (NN) [42,43,44] and deep neural network (DNN) [45]. The performance of the developed algorithms has been evaluated in terms of mechanical properties of the sensors and the accuracy of the applied algorithms under actual and realistic wearing test conditions. It has been proved that the textile sensors are extremely thin, lightweight, sensitive, and thus highly flexible and cause no harm, irritation or allergies to the skin.

Through controlling the pressure on the squeezing machine, we obtained fabric sensors with uniform final resistances and low migration of CNT powders on PET/SP fibers after stretching. Based on that, we suggest the possibility of mass production of these fabric sensors with an easy combination of sensor fabrication and machine learning algorithm models.

## 2. Materials and Methods

### 2.1. Materials

This research used a PET/SP fabric with a polyethylene terephthalate/spandex ratio = 76/24 (item 16043A, 341 g/YD, 262 g/SQM, from SNT Co. Ltd., Seoul, South Korea). The raw powder single-walled carbon nanotubes (SWCNTs) were obtained from KH Chemical Co. Ltd. (Seoul, South Korea). These SWCNTs were treated by a laboratory grade acid solutionn. The stirring machine, ultrasonication machine, auto dipping padding machine and two-way drying machine were sourced from Daelim Starlet Co. Ltd. (Seoul, South Korea). All other electronic components such as the Bluetooth module, microprocessor, lithium battery, etc. were used as purchased.

### 2.2. Methods

#### 2.2.1. Textile Sensor Fabrication

PET/SP fabrics, known for their exceptional elasticity, were prepared by co-weaving spandex with polyester. A small amount of spandex is used in the final fabric so that it may retain most of the look and feel of PET fibers. The PET/SP fabric is very resilient and can withstand a good deal of wear and tear, is waterproof and shows less wrinkling. These attributes make this spandex fabric widely applicable in industry to produce products such as clothing, household furniture, industrial fabrics, etc. The structure of the PET/SP fabric is composed of conventional PET/SP multifilament yarns with high elasticity and recovery. These fibers could be converted into conductive fibers via coating, padding, and surface treatment.

**Figure 1:**
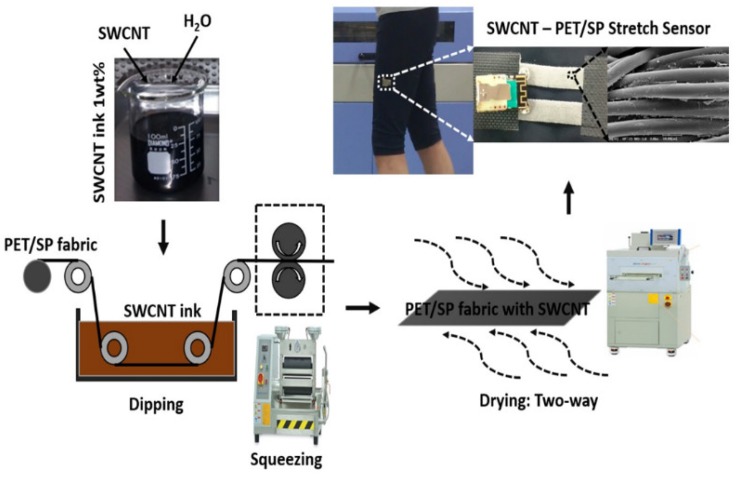
Summary of the fabrication process and application of SWCNT stretch sensors.

In order to fabricate the fabric sensor (Figure 1), carbon powder ink was applied by water-based single-walled carbon nanotube (SWNT) solution with nanotubes with 1.0–1.3 nm diameter and 0.1 wt.% concentration. The SWCNT powder was treated by acid solution (HNO_3_:H_2_SO_4_ = 3:1), dispersed in H_2_O, sodium dodecylbenzenesulfonate (SDBS), and ultra-sonicated (2 h, 19.990 Hz) in a stirring machine (60–80 °C, 1000 rpm, 24 h). The PET/SP fabrics were prepared and immersed in SWCNT ink within the bath of the automatic dipping padding machine. The impregnating process would maintain the conditions that allow the SWCNT particles to penetrate well (pressure roll speed: 1.0 m/min, air cylinder pressure: 3 bar (0.3 MPa) overpressure). This process would make the SWCNT particles adhere to the fabric surface after dipping and squeezing. After that, the two-way drying machine was used in order to get rid of the excess water in the fabrics. The drying conditions were optimized at the time of drying: 1–3 min, the range of temperature: 180–200 °C, and the speed of circulation fan: 1500 rpm. Finally, the fabric was maintained for 3–5 h under normal room temperature conditions. The fabric sensors were then cut to form smaller specimens for further experiments.

#### 2.2.2. Human Motion Analysis

**Figure 2:**
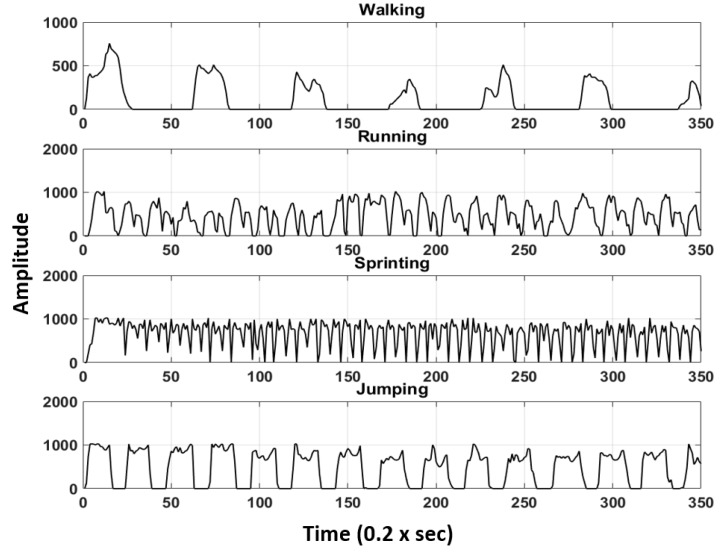
Types of human motion signals.

Actual muscle pants equipped with the fabricated textile sensor have been prepared for wearing test including motion analysis. During the test, three participants (Table 1) were asked to wear the smart muscle pants while moving. Four types of predefined motions are shown in Figure 2. The processing circuit digitized and sent motion data signals, and transmitted them via Bluetooth to a mobile phone in real time.

The method used to monitor activities is based on the relationship between the mechanical and electrical properties of the constituent conductive fabrics. Using a voltage divider circuit, the resistance variation has been converted into a voltage variation. Data based on the voltage was sampled/digitized and thus converted into the digital values. For resolution reason, mathematical mapping of voltage values between 0 to 3.7 volts into digital values between 0 to 1023 (3.7/1023 = 0.0037 V or 3.7 mV per unit) has been made by precalculating the actual data. It was calculated to take about 0.01 s (10 ms) to read an analog signal input, so the maximum reading speed is about 100 times per second. Motion data signals would be analyzed in order to generate three input parameters such as the average amplitude (AMP), standard deviation of the amplitude (STD), and the average cycle (CYC) for further processing.

**Figure 3:**
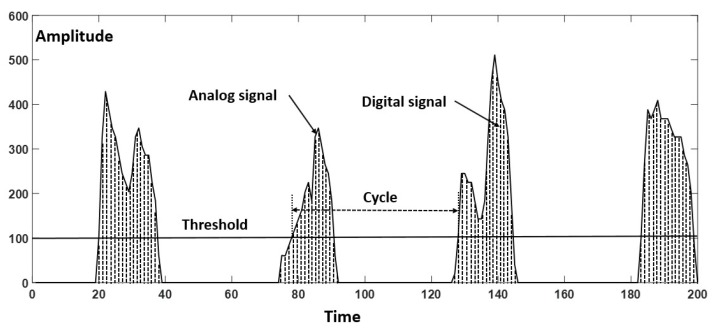
The parameters of the signal.

- Average amplitude: The average amplitude (AMP) is a commonly used term to indicate the magnitude of a periodic signal and determined by the ratio between the sum of the magnitudes of all instantaneous values and the number of considered instantaneous values. Considering a real signal as shown in Figure 3, A_1_, A_2_, A_3_, etc. are the magnitudes of the signal at instants 1, 2, 3, etc., respectively. The AMP is calculated as follows:
  (1) $$\;\mathrm{AMP}=\;\frac{A_{1}+A_{2}+A_{3}+\ldots +A_{n}}{n}\;$$
- Standard deviation of the amplitude: Standard deviation (STD) is a measure of the dispersion of data from its mean. It is calculated as the square root of variance by determining the variation between each data point relative to the mean. A low STD indicates that the data points tend to be close to the mean of the set data, while a high STD indicates that the data points are spread out over a wider range of values. Besides the average of amplitude, the STD evaluates the other aspect of the signal:
  (2) $$\;STD=\;\sqrt{\frac{\sum_{i=1}^{n}{\left|A_{i}-\bar{A}\right|}^{2}}{n-1}}\;$$
  where A_i_ represents an individual value, $\bar{A}$ represents the mean value, and n represents the total number of values.
- Average cycle: This is the most important parameter for the motion classification method proposed in this research. In the general fields of science and life, the cycle is defined by the shortest period in which an action is repeated. Average cycle (CYC) includes process time, during which a unit was acted upon to bring it closer to an output, and delay time, during which a unit of work was spent waiting to take the next motion. The CYC could be calculated through a threshold as shown in Figure 3.

#### 2.2.3. Machine Learning Models

**Figure 4:**
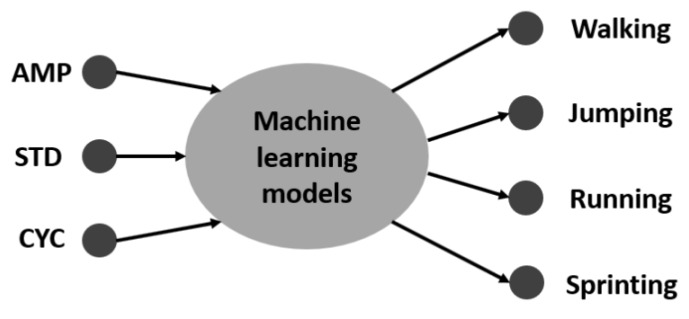
Structure of the models.

**Figure 5:**
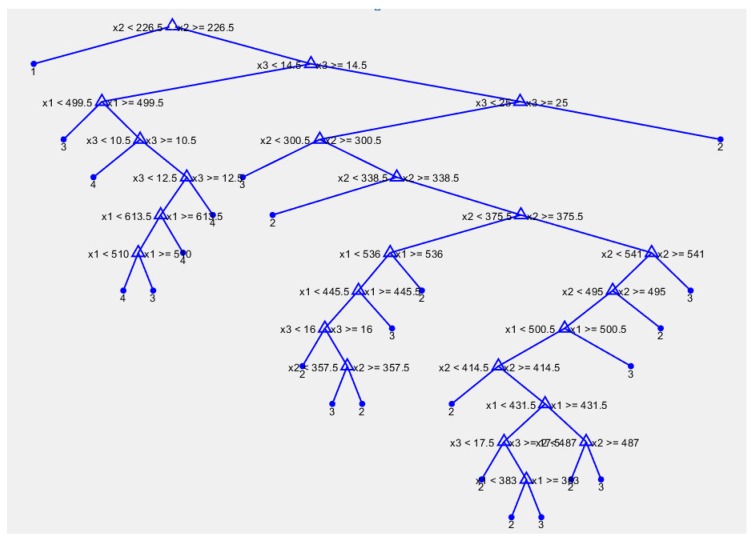
The graphical display of one tree in the RD model.

**Figure 6:**
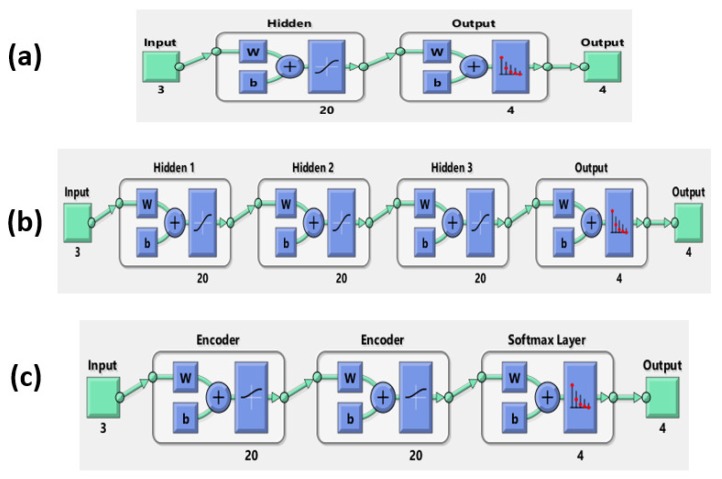
Structure of the neural network models: (**a**) One hidden layer, (**b**) Multi-hidden layers, and (**c**) Autoencoder.

The human motion dataset consists of 400 motion samples annotated in four classes such as walking, jumping, running, and sprinting. Using the ‘cvpartition’ [46] function in the MATLAB software, we split the dataset by assigning 75% to the training set and 25% to the testing set [31,47,48]. This research considered some machine learning models such as random forest (RD), support vector machine (SVM), one-hidden layer neural network (ANN), multi-hidden layers neural network (MANN), and autoencoders neural network. The main structure of these models is shown in Figure 4. Because the target of the study is easy to construct and quick to apply in a realistic product, we implemented the models based on MATLAB 2017b software, including: RD [49], SVM [50], ANN [51], MANN [51], and AE [52]. RD constructs a multitude of decision trees during the training time. Then, the final prediction is calculated by considering the high voted result predicted by each outcome tree [53]. Figure 5 shows one tree in the RD model. Triangle nodes are used as the splitting nodes and the bold dots are decisions of this tree. SVM looks for the optimal separating hyperplane between the classes by maximizing the margin between the classes’ closest points [53]. Parameters of the implemented multiclass model for SVM are shown in the Table 2. The implemented model used SVM binary learners, and a one-versus-one coding design [50]. Artificial neural networks (ANNs) are computing systems inspired by the biological neural networks that constitute the human brain. It is composed of multiple nodes connected with coefficients (weights) which constitute the different neural structures (one-hidden layer (ANN), multi-hidden layers (MANN), etc.) in order to perform certain specific tasks [53]. AE is special neural network structure based on the efficient coding. The encoder maps the input to a hidden representation and the decoder attempts to map this representation back to the original input [53]. As shown in Figure 6, the fabricated neural networks have different numbers of hidden layers, but each hidden layer has 20 neurons. In particular, the AE model has a softmax layer in order to get four predictions in the output.

## 3. Results and Discussion

### 3.1. Structure of the Stretch Textile Sensor

**Figure 7:**
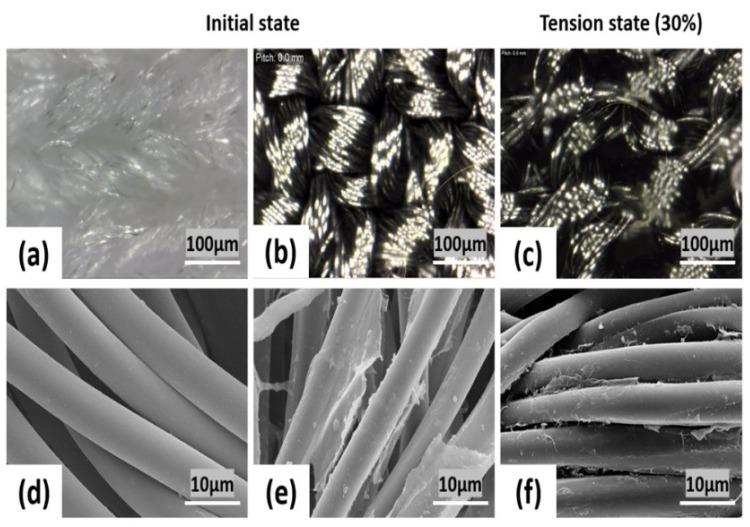
Surfaces of the fabricated sensors: (**a**) untreated, (**b**) treated, (**c**) treated and stretched (under lower magnification), (**d**) untreated, (**e**) treated, and (**f**) treated and stretched (higher magnification).

Scanning electron microscopy (SEM) was employed to characterize the morphological changes of the PET/SP fabric stretch sensors at different steps of the synthesis of the conductive fabric through the present approach. Figure 7 shows SEM images of the standard PET/SP fabric with the magnified view showing no coating on the fiber and the PET/SP fabric coated with SWCNT. The figure shows the surface morphology of PET/SP fabric at high and low magnification, in its initial state and tension state (30%), respectively. The diameter of the filaments is about 10 µm and appears loosely twisted with ample free space between the microfiber bundles. The particles could be observed in the form of a thin coating, and stuck randomly onto PET/SP fibers with a 80% coating rate.

The method for recognizing specific motion is strongly based on the relationship between the mechanical and electrical properties of the sensor fabrics. The resistance would change according to stretching or releasing by a responsive crack propagation mechanism. The cracks originate and propagate in the thin conductive layers coated on the PET/SP fibers during continuous mechanical stretching. They are released under the accommodated stress at the stress-concentrated areas and recover to their initial states after releasing the stretch force imposed on the fabrics. Edges of the cracks would reconnect at this point, ensuring complete recovery of the electrical resistance. The performance of the stretch sensor will be extremely sensitive and flexible based on this mechanism.

### 3.2. Stretchability (Yield Point) and Sensitivity (Gauge Factor)

**Figure 8:**
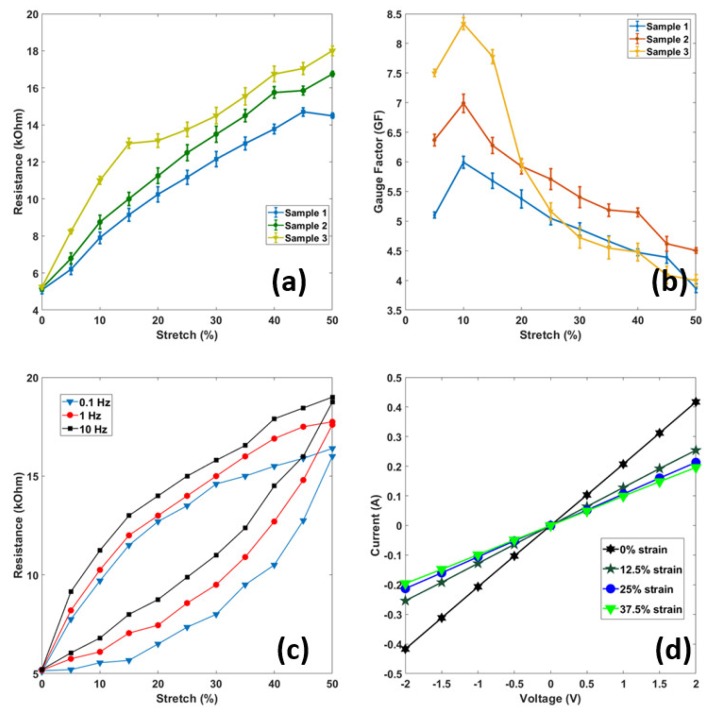
Characteristics of the sensors (I): (**a**) Stretch-ability, (**b**) Gauge factor, (**c**) Hysteresis, and (**d**) Current-Voltage curves.

The stretchability of a stretch sensor depends on the material of construction used, micro/nanostructures, and the fabrication process used in the study. Figure 8a shows the resistance- stretch relationship of three sensor samples. The structure of the PET/SP fabric is one of the main reasons for the high stretchability (yield point, ε_y_ ≈ 50%) of the resistive type sensor reported in this research. If the stretch is applied beyond a certain amount (ε > 50%), the PET/SP fabric would yield and the fabric will lose its sensing capability. The stretchability ensures a wide range of stretch sensing, enough for realistic applications. As shown in Figure 8b, the sensitivity or gauge factor (GF) of the three stretch sensors is defined as the ratio of a relative change in resistance (ΔR/R) and stretch (ε), and could be written as GF = (ΔR/R)/ε and ε = ΔL/L. It is clear that the resistance increases as the stretch increases, and viceversa. For the fabricated stretch sensor, the value of GF depends mainly on the SWCNT nanostructure. The results show that the GF ranges from 4.1 to 8.5, and depends on the stretch ratio (%). Based on the calculated GF values, the stretch sensor is sensitive and suitable for the applications in this research.

### 3.3. Current-Voltage (I-V) Curves

The I-V curve is one of the important parameters for characterizing stretch sensors. Figure 8d shows a set of graphical curves which are used to define the operation of the sensor under different static stretches from 0–37.5% within the system. The applied voltage from −2 V to 2 V indicated the resistance of stretch sensor was constant with an Ohmic behavior. The slope of the I-V curves reduces with an increase of applied stretch, from 0%–12.5%–25% and 37.5%, indicating that an increase in applied stretch led to an increase in the sensor’s resistance.

### 3.4. Hysteresis

Hysteresis is defined as a behavior whose output does not only depend on the current input but also on the history of the input. The hysteresis becomes important when the stretch sensor is used in dynamic applications such as human motion monitoring, ECG monitoring, healthcare, etc. Hysteresis behaviors are mainly caused by the elastic properties of PET/SP fabric, the interaction between SWCNT and PET/SP fibers, as well as the reconnectability of the thin coatings after release of the applied stretch. Strong interfacial binding between the SWCNT nanostructures and PET/SP fibers gave the good stretch sensing performances. The hysteresis behaviors of three frequencies are shown in Figure 8c, indicating a linear rise in resistance when applying stretch and a small hysteresis.

### 3.5. Response and Recovery Time 

Response time is the time taken to initially react to a given input. The response delay in the sensors is mainly caused by the viscoelastic nature of the PET/SP fabric. The experimental results showed a response time of 200 ms at ε = 30%. Recovery time is another important parameter of the stretch sensor in order to evaluate the performance in dynamic applications. The recovery time of this fabricated stretch sensor is 220 ms at ε = 30%. The recovery time is affected by the friction force and the reconnectability between the SWCNT coatings and the PET/SP fibers. The fast self-recovery process of the SWCNTs ensures rapid recovery of the electrical property of the stretch sensor and avoids the degradation of the device performance during large deformations.

### 3.6. Durability

**Figure 9:**
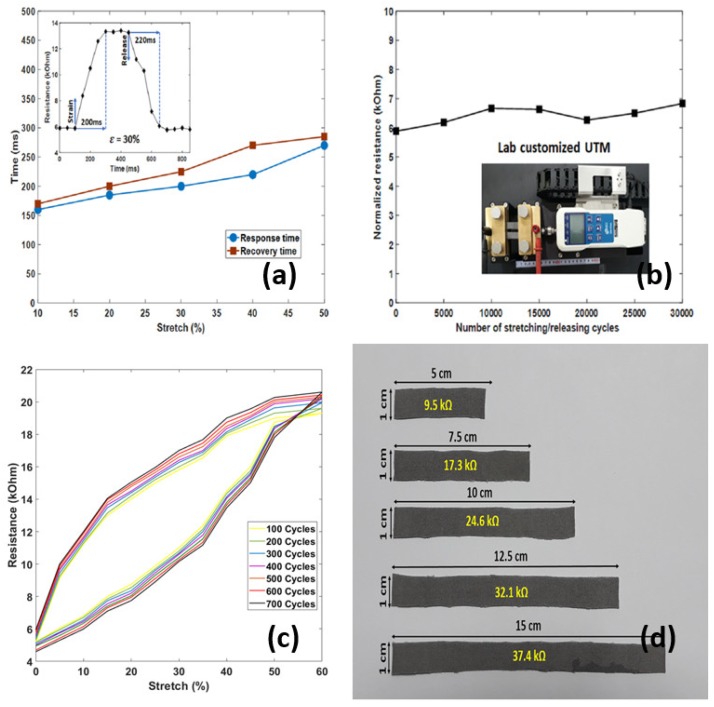
Characteristics of the sensors (II): (**a**) response-recovery time; (**b**) resistance of sensors after 30,000 cycles of dynamic tension test (30%); (**c**) stretch-resistance of 700 cycles of dynamic tension test (30%), and (**d**) resistance of the sensors in five different samples.

The dynamic durability is the stable electrical functionality and mechanical integrity of the stretch sensor during its stretching/releasing cycles. This parameter depends on the fatigue and plastic deformation of the PET/SP fibers under high stress which causes damage to the fibers (PET) and the sensing nanomaterials (SWCNTs). The durability was tested in the laboratory using a customized UTM for the tension tests. The resulting fabric surface was intact after 30,000 stretching/releasing cycles, that means repeated stretch under 30% would not affect the sensor performance within 30,000 cycles. Through controlling the pressure on the squeezing machine, the uniform resistance of samples is shown in Figure 9b–d. All samples have resistance changes of less than 10% after 30,000 cycles of 30% tension. Especially, Figure 9c showed a low migration of CNT powders on PET/SP fibers during the stretch/release cycles. That result clearly reveals that the dynamic durability was enough for practical applications.

### 3.7. Human Motion Classification

The application capability of the fabricated stretch sensor would be evaluated by the testing on four human motions such as walking, jumping, running, and sprinting. The experimental environment of motions is in a straight corridor. Participants was asked to wear the smart muscle pants while moving in the straight corridor (a total length of 50 m). Characteristics of motions are shown in Table 3, included: velocity (m/s), step size (m), and frequency (Hz).

**Figure 10:**
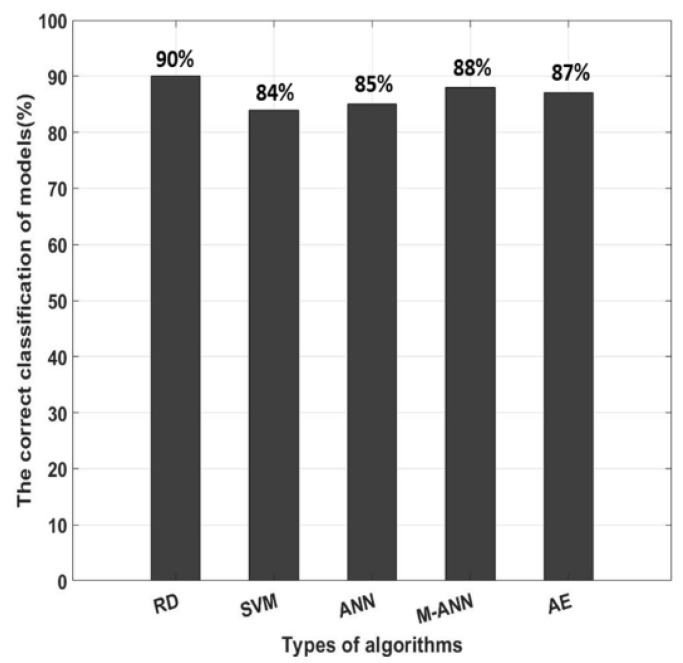
Comparison of the correct classification motions of the models (RD, SVM, ANN, M-ANN, AE) with actual motions.

The result of the experiments are shown in the comparison between the output of the system and the actual motion. Statistical indices, including percent accuracy and confusion matrix are two important elements to evaluate the computational efficiency of this research. The accuracy is determined by Equation (3):

(3) $$\;A=\frac{TP+TN}{TP+TN+FP+FN}\;$$

where *TP*, *TN*, *FP*, and *FN* represent the number of true positives, true negatives, false positives, and false negatives, respectively. In Figure 10, the five algorithms obtained a mean performance accuracy of 90% with the random forest, 84% with the support vector machine, 85% with one hidden layer neural network, 88% with multi-hidden layers neural network, and 87% with autoencoder. The accuracy of the random forest algorithms (90%) shows that there is a good agreement between the measured and classified values.

**Figure 11:**
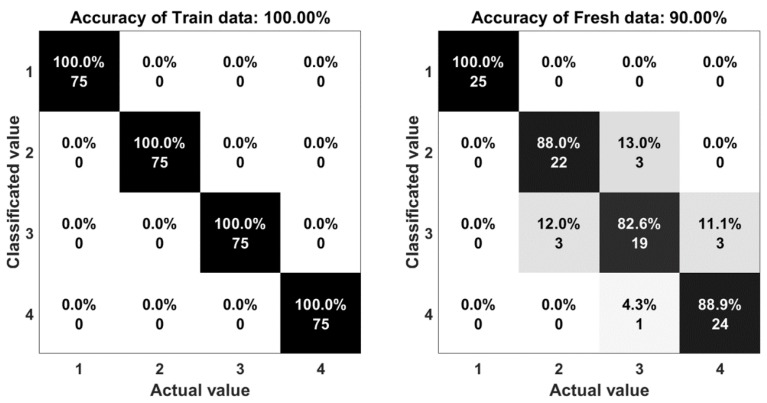
The accuracy of the random forest algorithm.

**Figure 12:**
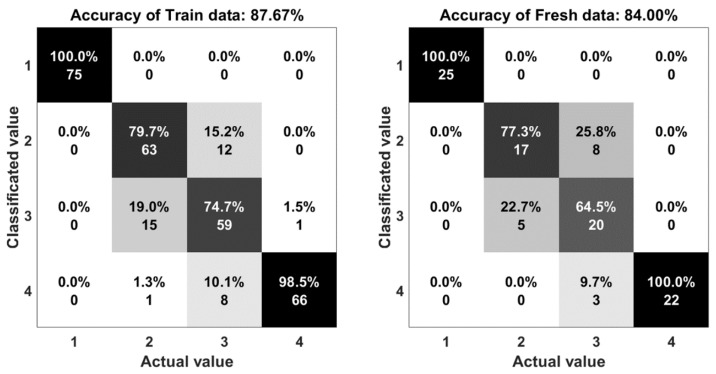
The accuracy of the support vector machine algorithm.

**Figure 13:**
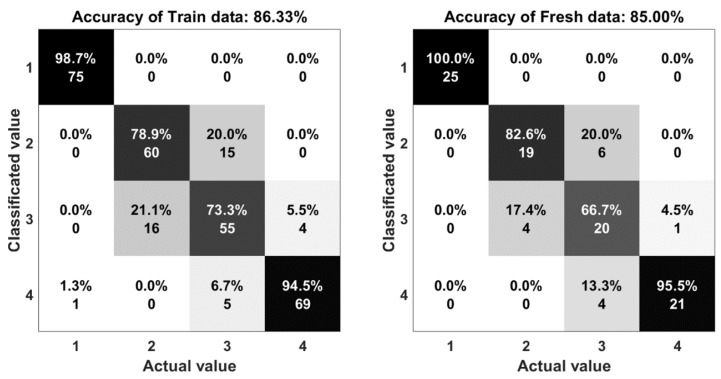
The accuracy of the one-hidden layer neural network algorithm.

**Figure 14:**
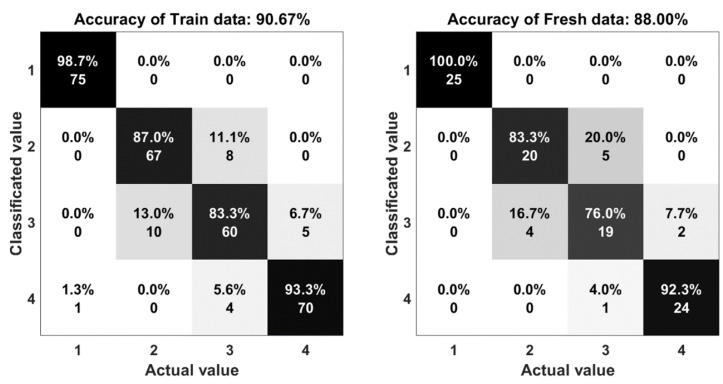
The accuracy of the multi-hidden layers neural network algorithm.

**Figure 15:**
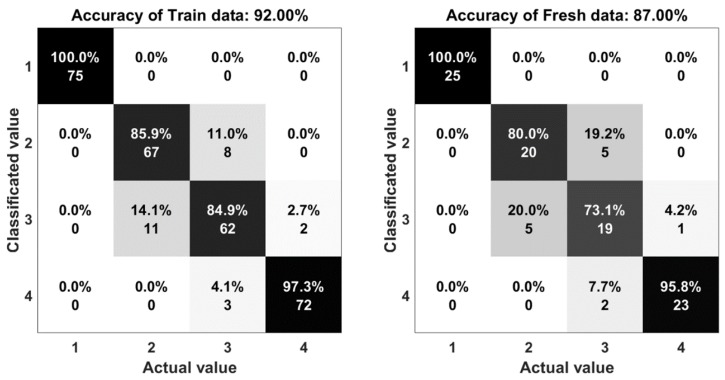
The accuracy of the autoencoders algorithm.

The confusion matrix or error matrix is a specific parameter in the field of statistical classification of machine learning. This is a specific table layout that allows visualization of the performance of algorithms. Each row of the matrix represents the instances in a classified value while each column represents the instances in an actual value. The confusion matrices of the algorithms are shown in Figure 11, Figure 12, Figure 13, Figure 14 and Figure 15. Here number 1 represents walking, number 2 represents jumping, number 3 represents running and number 4 represents sprinting. The diagonal elements represent the number of cases for which the classified motion is equal to the actual motion, while off-diagonal elements are those that are mislabeled. The higher the diagonal values of the confusion matrix the better, it indicates many correct classifications. Accordingly, it is clear that the walking and sprinting motions are easy to classify with all algorithms such as RD(100–88.9%), SVM(100–100%), ANN(100–95.5%), M-ANN(100–92.3%), and AE(100–95.8%), respectively.

However, the jumping and running motions are easy to confusing such as RD(88–82.6%), SVM(77.3–64.5%), ANN(82.6–66.7%), M-ANN(83.3–76%), and AE(80–73.1%). Especially, the precision of the running motion in the support vector machine and the one hidden neural network algorithms are lowest. From the matrices, the running motion was confused with the jumping motion in SVM model (25.8%), ANN and M-ANN models (20%). The ratio of this confusion is still high in AE model (19%). The best ratio is 13% of the RD model. Besides that, the jumping motion was easy confused with the running motion in SVM model (22.7%) and AE model (20%). This ratio in the ANN model and M-ANN model are almost same (16–17%).

The research has demonstrated that the random forest, multi-hidden layers neural network and the autoencoders algorithms were superior to the support vector machine and the one hidden layer neural network in terms of classification accuracy in this realistic application. The results obtained from the random forest and the multi-hidden layers neural network algorithms were similar in terms of classification rate, and the random forest was marginally better than the multi-hidden layers neural network. Based on that result, we suggest the RD model be applied in real applications for mass production. The main reason is the high accuracy of the RD model. In addition, the algorithm of the RD model is easy to understand and it is supported in the MATLAB tools.

## 4. Conclusions

This research has developed a complete combination of the wearable application based on SWCNT-PET/SP and machine learning models to analyze sensing signals from a real product. The research emphasized the possibility to bring the product from experimental concept to daily life with high economic efficiency, simply and quickly. The fabrication process of the stretchable and flexible stretch sensor is simple and the performance of the monitoring model was enhanced by machine learning algorithms. Based on the statistical indices, the high accuracy demonstrated that this system could be applied as an intelligent device for recognizing human motions in real time. However, the research still has some limitations. The high variation of the sensor response causes bias in the final results. We suggest using vacuum drying in the fabrication of the sensors. This process can create a strong connection between SWCNTs and PET/SP fibers. These are future directions of the project.

## Figures and Tables

**Table 1:** Information about participants.

Age (year)	Gender	Weight (kg)	Height (m)
28	Male	55	1.67
26	Male	62	1.70
32	Male	65	1.72

**Table 2:** Parameters of the multiclass model for support vector machines.

Name	Characteristic
Response Name	‘Y’ (Output)
Categorical Predictors	[none]
Class Names	[‘Walking’ ‘Jumping’ ‘Running’ ‘Sprinting’]
Score Transform	‘none’
Binary Learners	{6 × 1 cell}
Coding Name	‘onevsone’

**Table 3:** Characteristics of motions.

Characteristic	Velocity (m/s)	Step Size (m)	Frequency (Hz)
Walking	1.2	0.35	1.7
Running	3.2	0.45	2.4
Sprinting	5.0	0.7	3.0
Jumping	1.5	0.75	2.0
